# Group Telerehabilitation to Improve Balance and Mobility in Patients After Stroke Performed at Home: A Feasibility and Pilot Study

**DOI:** 10.3390/healthcare14010129

**Published:** 2026-01-04

**Authors:** Metka Močilar, Nataša Bizovičar, Urška Puh

**Affiliations:** 1 University Rehabilitation Institute, Republic of Slovenia—Soča, 1000 Ljubljana, Slovenia; metka.mocilar@ir-rs.si (M.M.); natasa.bizovicar@ir-rs.si (N.B.); 2 Department of Physiotherapy, Faculty of Health Sciences, University of Ljubljana, 1000 Ljubljana, Slovenia

**Keywords:** telerehabilitation, remote training, stroke, chronic phase, balance, mobility

## Abstract

**Background/Objectives**: Telerehabilitation is intended for remote treatment, but studies on group training after stroke performed in the patient’s home have not yet been conducted. The purpose of this study was to evaluate adherence rates, safety, usability and enjoyment and preliminary clinical effects of home-based remote group training for balance and mobility in chronic stage after stroke. **Methods**: Community-dwelling patients in chronic stage after stroke who walked independently and had mild balance deficits participated. Over a 6-week period, they completed 60-min sessions of balance and mobility training twice a week in a group from their home. Adherence rates, adverse events and technical problems were recorded. Participants’ satisfaction was assessed using Modified Physical Activity Enjoyment Scale. The primary outcomes were Mini-Balance Evaluation Systems Test (mini-BESTest), 5TSTS and 10MWT and secondary outcomes were limits of stability, weight-bearing symmetry and Activities-Specific Balance Confidence Scale (ABC Scale), measured before, immediately after and six weeks after remote group training. **Results**: Participants expressed a very high level of satisfaction with training. There were no adverse events and dropouts, but some minor technical challenges. Results showed significant improvements in primary outcomes (mini-BESTest, 10MWT fast walking speed, 5TSTS; all *p* < 0.001), however there were no significant improvements in secondary outcomes (weight-bearing symmetry, limits of stability and ABC Scale). All improvements persisted six weeks after training. **Conclusions**: Remote group training at home is feasible, safe and efficient to improve balance and mobility in patients in the chronic phase after stroke.

## 1. Introduction

Motor impairments after stroke can manifest as limitations in mobility and balance control [1]. Regaining mobility is a priority for stroke patients as it is important for independence in activities of daily living [2]. In about one-fifth of chronic post-stroke patients, mobility status deteriorates significantly one year after stroke [3]. Impaired balance control and mobility problems are risk factors for falls in community dwelling stroke survivors [4], with ambulatory survivors more likely to fall than those with more limited mobility [5]. Even individuals in the chronic phase after a mild stroke show persistent balance limitations and have an increased risk of falls [6].

Targeted exercise therapy programs, specifically, balance and/or functional weight-shifting training, have been shown to be successful training regimens to improve balance capacities in the chronic phase post-stroke [7]. As studies that have assessed improvement in the International Classification of Functioning, Disability and Health (ICF) [8] component of body functions and structures are rare, it is not yet clear whether improvement in balance in the chronic phase occurs only at the ICF activities component [7]. This knowledge would allow a distinction between motor recovery and compensatory strategies [9,10]. Repetitive sit-to-stand training can have a positive effect on the time required and weight-bearing symmetry during sit-to-stand in patients who were already able to stand up independently [11]. For people with long-term post-stroke mobility problems, lifelong maintenance of physical fitness and promotion of physical activity are required [12]. Therefore, there is a need to develop new therapeutic approaches such as telerehabilitation.

Telerehabilitation is intended for remote treatment using telecommunication devices [13,14]. It allows for wider geographical and financial accessibility of rehabilitation services, earlier discharge from hospital and continuation of rehabilitation [15,16,17]. In patients in the acute [13,18,19] and chronic [20] phases post-stroke, it has been confirmed that remote individual home-based physiotherapy is just as effective as conventional (in-person) physiotherapy in improving functional balance [13,18,19,20], mobility [19] and activities of daily living [13,19,20] and similarly effective in improving quality of life [19]. Patient satisfaction with individual remote physiotherapy was also similar [20]. To date, only two randomized controlled trials (RCTs) of remote group training, conducted in healthcare facilities, have been published. Remote group training in addition to standard care in an environment that resembled the patient’s home [21] and remote group training in a healthcare facility alone [22], that also included balance training, have been shown to improve functional balance and mobility after stroke [21,22]. In the first study [21], participants trained in their individual hospital rooms, while in the second study [22] all participants trained in one space. Recent clinical practice guidelines [23] recommend telerehabilitation to improve the balance of stroke patients. Sufficient physical, cognitive and communication abilities are required to participate in remote balance and mobility training, and these must be higher for group training. However, the requirements for such training are not yet clearly defined. Considerations of requirements need to be made to ensure accessibility and engagement in telerehabilitation post-stroke [23,24].

The training protocol of this study was prepared based on known evidence on functional training regimes to improve balance in patients after stroke [7,11], suitable to be performed remotely, considering the identified gaps of the previous remote group training studies [21,22]. The purpose of this study was to investigate the feasibility (including adherence rates, safety, usability and enjoyment) of remote group training in stroke patients, who were able to walk independently and had mild balance deficits. The purpose was also to estimate the preliminary effects of home-based remote group training for balance and mobility.

## 2. Materials and Methods

This was a prospective feasibility and pilot study, registered at ClinicalTrials.gov (NCT06365463).

### 2.1. Participants

A sample of convenience was recruited from the list of patients who had completed a regular inpatient rehabilitation at our institution and through members of the Association of Patients with Cerebrovascular Diseases. Adults (18 to 70 years) in the chronic phase of ischemic or haemorrhagic stroke (>6 months) were eligible for participation, if they were able to walk independently on level ground or on all surfaces with or without a walking aid (Functional Ambulation Classification 6/6), had mild balance deficits (Mini-Balance Evaluation Systems Test—mini-BESTest 14-23/28) [25] and sufficient cognitive and communicative abilities for participation. Participants were excluded, if they had a stroke in the brainstem or cerebellum, additional neurological condition and/or musculoskeletal impairments that would interfere with the training, or advanced heart failure (New York Heart Association functional classification III–IV). To participate in the study, participants had to have access to a personal computer with a camera and internet connection. The study did not include a control group, in accordance with the exploratory nature of feasibility and pilot research. The study complied with the Declaration of Helsinki and was approved by the National Medical Ethics Committee of the Republic of Slovenia (No. 0120-259/2022/3). All participants gave their written informed consent to participate.

### 2.2. Protocol

**Figure 1:**
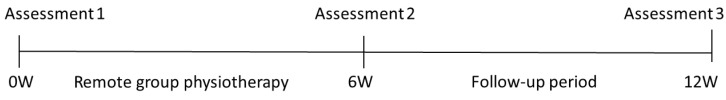
Study design: participants were assessed before and after home-based remote physiotherapy and after a six-week follow-up. Both the training and the follow-up phase lasted six weeks.

Participants took part in remote one-hour balance and mobility training sessions, performed biweekly for six weeks, followed by a six-week follow-up period (Figure 1). The protocol was conducted in two consecutive groups of participants.

The participants trained from home. Before each training session, participants received an email reminder. To increase adherence, verbal promotion and motivation was given at the end of training sessions. A physiotherapist helped with login problems and conducted the training via the online tool Zoom on the participants’ personal computers using a live videoconference. An additional physiotherapist helped with monitoring participants during training sessions, giving them instructions on correct movements/exercise execution (encouraging movement symmetry and use of the stroke-affected body side). This allowed for controlling movement quality and to increase the difficulty of the exercise. Caregivers were present in case help was needed to operate the technology and to ensure safety supervision during the training. Each session included a warm-up, 11 function-specific exercises to improve balance and mobility (e.g., sitting forward reach, standing up and sitting down, weight transfers, heel lifts, stepping in different directions) and stretching to cool down. Each function-specific exercise had 4 levels of difficulty (higher number of repetitions, smaller base of support, added weights) (see Supplementary Materials File S1). All participants started with the first difficulty level. Their progress depended on the individual baseline assessment and the improvement in performance observed. If absent, participants were encouraged to perform the training session themselves using a video recording. During the follow-up period, participants were encouraged to continue training by themselves at least once a week using video-recorded sessions.

### 2.3. Assessment

All participants were assessed in the institution within two weeks before, within one week after the remote training period and within one week after a six-week follow-up (Figure 1).

Attendance at the remote training and the number of individual sessions using video recordings were documented. Adverse events and technical problems during or related to the training were documented. The participants’ satisfaction with the remote training was assessed using the modified Physical Activity Enjoyment Scale (mPACES) [26]. This is a five-item scale in which the score is given as a percentage. In addition, participants were asked whether they would continue remote physiotherapy, how often per week they would prefer it and what was their favorite and most difficult exercise.

The primary outcome measures of balance and mobility were the mini-BESTest [25], the 5 Times Sit-to-Stand (5TSTS) test [27] and the 10-m walk test (10MWT) [27]. The 10MWT was used to measure comfortable and fast walking speeds, while the 5TSTS assessed sit-to-stand capacity. The secondary outcome measures of balance were limits of stability (LOS), weight-bearing symmetry and the Activities-Specific Balance Confidence Scale (ABC Scale) [27]. LOS and weight-bearing symmetry were measured using a force plate (Equio; Kinestica d.o.o., Ljubljana, Slovenia). Force plate measures have been shown to demonstrate high test-retest reliability in patients in the sub-acute phase post-stroke, particularly mean center of pressure speed and weight-bearing asymmetry [28], while also exhibiting moderate concurrent validity with clinical scales like the Berg Balance Scale [29].

### 2.4. Analysis

Average LOS in four directions and in the direction of the stroke-affected side were used to determine the change, excluding patients in whom both sides of the body were affected by the stroke. To determine improvement in weight-bearing symmetry, the absolute difference between the left and right sides was used. In addition, the improvement in weight-bearing on the stroke-affected lower limb (%) was assessed, excluding patients with bilateral involvement. Missing data were excluded from the analysis.

Data were tested for normality using the Shapiro–Wilk test. Repeated measures ANOVA or Friedmans test and post hoc paired *t*-test or Wilcoxon’s signed rank test with Holm correction for two comparisons were used to test for differences between assessment 1–2 and 2–3. Level of significance was set at the value alpha ≤ 0.05. To quantify the magnitude of change in balance performance, an effect size was calculated for the mini-BESTest using Cohen’s d for paired samples. The minimal detectable change (MDC) of 3 points for the mini-BESTest [30,31], was used as a benchmark for interpreting clinical relevance. R 4.3.1. (Bell Laboratories, Murray Hill, NJ, USA) was used for the statistical analysis.

## 3. Results

### 3.1. Participants

A total of 19 participants were initially recruited for the study. Six individuals were subsequently excluded based on predetermined criteria: three due to not reaching and two due to surpassing the limits of Mini-BESTest (14–23 points) and one due to an unstable health status. Consequently, a final sample of 13 participants was included in the remote training program. Their ages ranged from 43 to 70 years. The time since stroke ranged from 11 to 326 months (Table 1). Participants performed the study protocol in two consecutive groups of eight and five participants. One participant dropped out at follow-up due to a health complication unrelated to this study. For raw data of the study, see the article Supplementary Materials File S2.

At baseline, mini-BESTest scores ranged from 14 to 22; seven participants achieved the fourth (14–18 points) and six the fifth (19–23 points) out of six balance categories [32]. Seven participants had an increased risk of falls (mini-BESTest ≤ 17 points) [30]. Eleven participants were fully ambulatory in the community (comfortable walking speed > 0.93 m/s) [33]. For balance and walking abilities at baseline, see Table 2.

### 3.2. Feasibility

On average, participants attended 11.2 out of 12 (range 9–12) training sessions. Six participants were not always able to attend the live training sessions via videoconference due to illness or other commitments and therefore conducted 1 to 4 training sessions themselves using a video recording. During the follow-up period, the participants completed on average 5.5 (range 0–12) training sessions with the video recordings.

There were no adverse events, including falls, other health complications or side effects, during training or related to training. Technical issues in some training sessions were due to camera malfunctions for two participants (one participant’s camera did not work and another participant had problems positioning the camera) and two participants’ home internet connection was interrupted in one session. On one occasion, the physiotherapist’s computer crashed, delaying the training by 10 min. No intervention by the caregivers was needed to ensure safety during remote training. The technical issues stated above were solved with the help of the caregivers.

The participants enjoyed the remote training and rated the mPACES at 92.1%. All wanted to continue with the remote group physiotherapy, on average twice a week (SD 0.6; range 1–3). Seven indicated that their favorite exercise was stepping in different patterns, while the most difficult exercise varied among participants.

### 3.3. Effects on Balance and Mobility

The mini-BESTest scores increased significantly after training and were maintained at follow-up (Table 2). The 5TSTS results and fast walking speed increased significantly after training and were maintained at follow-up. However, no statistically significant difference in comfortable walking speed was found between the three assessments (Table 2).

A high negative correlation was found between the improvement in fast walking speed and the time since stroke (ro = −0.68; *p* = 0.01). No other statistically significant correlations were found with improvement in other parameters.

When comparing the mean LOS measures of all four directions for all variables, there were no significant differences between the three assessment time points (Table 3). Post-hoc analysis of movement velocity (MVL) and direction control (DCL) also showed no improvement in the direction of the stroke-affected side. Three participants were unable to perform LOS 75% and were therefore not included in the analysis. Similarly, there was no significant difference in weight-bearing symmetry (Table 4). No significant improvement was found in the ABC Scale scores either.

## 4. Discussion

The aim of our study was to investigate the feasibility and preliminary effects of remote group training in patients in the chronic phase post-stroke. Feasibility outcomes (safety, adherence rates and the usability of remote delivery) indicate that group telerehabilitation is a practical and acceptable approach for stroke patients who walk independently and have mild balance deficits. At the same time, the clinical findings revealed meaningful improvements in balance and mobility, with several participants surpassing the mini-BESTest, fast walking speed and 5TSTS MDCs. These preliminary results suggest that the intervention has potential therapeutic value and justify progression to a two-arm parallel-group RCT to compare the effects of the same training program performed remotely and in person. The strength of our study lies in the comprehensive assessment of feasibility and improvements in both ICF components, the activities, the body functions component and the rare example of the remote group home training. It should be noted that, similar to both previous studies [21,22], remote group training has been performed after participants completed their standard rehabilitation.

The sample size of this study is comparable to similar previous individual and group training studies using telerehabilitation [20,21,22]. Adherence was excellent, as all 13 participants completed the full volume of the training program. Although not all participants attended every live session, those who missed a live session successfully caught up by using the provided video recordings. No adverse events were noted, except for some minor technical issues (positioning of the camera, occasional disturbance of the internet access or computer malfunction), which were solved with the help of the caregivers. We found that participants had remarkably high levels (92%) of enjoyment during remote group training. This result is consistent with a previous study [20] on individualized remote and conventional training, in which patients in the chronic post-stroke phase reported comparable high levels (~80%) of satisfaction. We agree with the propositions [34,35,36,37] on group training as an effective motivational and engaging strategy in stroke patients. Within the group, we noticed valuable peer support, sharing of experiences, networking, self-encouragement and motivation to participate in the training.

In our study, the overall improvement in primary outcome measures was evident after six weeks of remote group training. Almost all activities assessed with the mini-BESTest, the 5TSTS and the 10MWT (fast walking speed) showed individual participants’ improvement. The group mean improvement in the mini-BESTest (of 2.9 points) after remote training reached a higher, fifth, category of balance ability (19–23 points) [32]. In addition, five participants exceeded the minimal clinically important difference of 4 points [38,39] and seven exceeded the MDC of the mini-BESTest of 3 points [30,31], indicating substantial individual progress. However, the mean improvement in the mini-BESTest score in our study was lower than the 4.1 and 6.2 points [39] achieved after conventional balance training. In both previous studies [31,39], patients received more training sessions than in our study and were in the subacute phase post-stroke. Similar to our study, patients in the chronic post-stroke phase improved their mini-BESTest score by 1.8 points after virtual reality balance and gait training [40]. In previous studies on remote balance training with patients in the chronic phase post-stroke, which were conducted as individual [20] and group training [22], an improvement in the Berg Balance Scale score was also reported.

For fast walking speed, five participants exceeded the MDC of 0.13 m/s [41]. The increased fast walking speed is likely due to an improvement in dynamic balance, as demonstrated by the mini-BESTest dynamic tasks mentioned earlier and 5TSTS. The increased fast walking speed is consistent with previous study [39] on the effects of conventional balance training in the subacute phase after stroke. However, in our study as well as in a previous study [42] with patients in the chronic phase post-stroke after conventional physiotherapy, the comfortable walking speed did not change. On the contrary, in many other studies, an improvement in comfortable walking speed (0.05–0.35 m/s) was found after conventional balance training in patients in the chronic phase post-stroke [43,44,45,46]. This is likely due to the limitations of (group) remote training, which cannot include walking balance exercises because of the camera-controlled spatial constraints and associated safety reasons.

The training program in our study included a high proportion of repeated sit-to-stand activities with increasing difficulty, which may explain the statistically significant improvement in 5TSTS after training (group mean decrease: 4.2 s). Six participants and the group average exceeded the MDC of 2.9 s [47]. Similar results have been reported in previous studies with patients in the chronic post-stroke phase after conventional balance training [48,49]. Improved balance is also known to influence 5TSTS outcome in stroke patients [50,51,52].

Remote training does not allow manual guidance for instruction, movement correction and safety. In our study, two physiotherapists constantly corrected the movement quality of the participants verbally, thus promoting symmetry and increasing the difficulty of the exercises individually. Therefore, we believe that the improvement is at least partly due to greater engagement of the stroke-affected side and its recovery of motor function (i.e., improved muscle strength and coordination of body movements). Since muscle strength or electromyography was not measured, we cannot state with certainty that the improvement is also not due to more effective compensation (i.e., greater involvement of the unaffected side) [9,10].

Importantly, improvements in the mini-BESTest, fast walking speed and 5TSTS persisted for at least six weeks after completion of the guided remote training, suggesting mid-term sustainability. The results are consistent with studies reporting long-term effects (12 weeks) on balance and fast walking speed after individual remote physiotherapy [13] and after Balance Master training [46] in the acute and subacute post-stroke phases, respectively.

However, for the secondary outcome measures—weight-bearing symmetry and LOS, which assess balance in the ICF body functions component—we found no significant difference or improvement. Similarly, no difference in symmetry was found after conventional balance training in the chronic phase post-stroke [53,54]. In contrast, interventions involving virtual reality training have shown a 3–4% improvement in weight-bearing symmetry [54,55]. Notably, these interventions included more training sessions than in our study and more accurate visual biofeedback through virtual reality, which has a positive effect on static and dynamic balance in the chronic post-stroke phase [42,56]. In addition, a study [57] assessing the effects of multicomponent training on LOS found a 0.4% increase in MVL.

While we found no significant improvements in balance confidence after training, there was a notable reduction (from seven to four) in the number of participants scoring below the ABC Scale threshold of 81.1% for fall risk. It is known that improving dynamic balance can reduce fear of falling [45], a trend supported by our results. For comparison, a study [54] reported significant improvements in balance confidence in the chronic phase post-stroke after both virtual reality balance training and conventional balance training. However, it should be noted that participants in the previous study [54] had lower baseline levels of balance confidence (52.1% and 59.6%) than the participants in our study and that their participants did not exceed the MDC threshold of 18.9% [58].

Although all participants in our study were in the chronic post-stroke phase, the range of time since stroke was large. In agreement with the study [42], which emphasized the influence of the chronic phase post-stroke on the extent of improvement and long-term effects, our results showed a high negative correlation between time since stroke and an increase in fast walking speed. This correlation is similar to the results of meta-analyses [59,60], which observed greater improvements in fast walking speed in patients in earlier post-stroke phases than in later phases. The observed decline in improvement may be attributed to the increasing prevalence of complications such as spasticity, soft tissue contractures and degenerative changes in muscles and joints over time [61,62]. These factors could potentially limit functional progress and contribute to the negative correlation result.

Since this study confirmed the safety of group remote training in patients with mild balance deficits, future studies may progress to assessing the feasibility of remote (group) training in patients post-stroke with moderate balance deficits. In this case, more rigorous baseline assessments, including risk of falls assessment and dual-task capacity, should be included, followed by a tailored exercise program and appropriate physical assistance.

Certain limitations of this study must be considered, most notably the small sample size and the absence of a control group, which limit the generalizability of the findings. However, a control group was not strictly necessary, as the primary objective was to establish the viability and technical refinement of the protocol before proceeding to larger-scale controlled trials. Furthermore, this sample size appears sufficient to evaluate the potential efficacy of this novel approach, while recognizing the need for cautious interpretation of group findings. Therefore, we also focused on interpreting individual participants’ improvements with regard to known MCIDs. An additional limitation of this study is that only mid-term sustainability was assessed; therefore, future studies should measure long-term effects, for example, three or six months after training.

Inclusion of a control group receiving standard care without the remote group training (or no physiotherapy program, which would be acceptable if participants completed their standard rehabilitation) is essential to confirm the internal validity of the findings, demonstrating that the observed improvements are directly attributable to the program itself. Furthermore, a second parallel RCT comparing the telerehabilitation program to a comparable group receiving the same training protocol delivered in-person is required to establish non-inferiority and determine if the remote delivery format is equal in efficacy and safety to traditional in-person training.

## 5. Conclusions

Remote group training at home is feasible, safe, enjoyable and a promising method for improving balance and mobility for chronic stroke patients who walk independently and have mild balance deficits. The mid-term individuals’ improvement, safety and high satisfaction of the participants support the potential of this method to be used as a valuable therapeutic approach in stroke rehabilitation. A future two-arm parallel-group RCT with a control group and/or a comparable group with the same training program delivered in-person using the calculated sample size is needed to confirm/validate the results of this study.

## Figures and Tables

**Table 1:** Participants’ characteristics: demographics, clinical characteristics and walking aids in the community (n = 13).

Characteristic	Value
Sex (n), male/female	4/9
Age (years), mean (SD)	56.0 (7.3)
Time since stroke (months), mean (SD)	76.1 (107.7)
Stroke-affected side (n), left/right/bilateral	4/6/3
Walking aid in the community (n):	
None/crutch/rollator	8/1/1
Ankle-foot orthosis	4

**Table 2:** Comparisons of clinical assessment results before and after training and at follow-up (n = 12).

Clinical Assessments	Mean ± SD	Range [Min–Max]	Time Effect		Comparison Between Two Assessments	
			F ^1^/χ^2 2^	*p*	T ^1^/Z ^2^	*p*
Mini-BESTest						
Baseline	17.4 ± 3.3	14–22				
6 weeks	20.3 ± 4.2	15–26	36 ^2^	<0.001 *	0 ^2^	0.002 *
12 weeks	20.8 ± 3.8	15–27			1.07 ^1^	0.309
10MWT_Fws						
Baseline	1.45 ± 0.30	1.16–2.08				
6 weeks	1.54 ± 0.29	1.14–2.07	25 ^2^	<0.001 *	12 ^2^	0.019 *
12 weeks	1.60 ± 0.27	1.3–2.11			1.71 ^1^	0.115
10MWT_Cws						
Baseline	1.06 ± 0.17	0.83–1.38				
6 weeks	1.08 ± 0.13	0.88–1.29	0.681 ^1^	0.513		
12 weeks	1.13 ± 0.19	0.92–1.26				
5TSTS						
Baseline	17.0 ± 7.4	10.4–38.4				
6 weeks	12.6 ± 2.4	8.8–16.6	21.8 ^2^	<0.001 *	73 ^2^	0.004 *
12 weeks	12.7 ± 2.9	8.43–17.01			0.21 ^1^	0.841
ABC Scale						
Baseline	80.4 ± 15.7	46.9–96.6				
6 weeks	84.5 ± 15.2	51.3–95.9	0.863 ^1^	0.431		
12 weeks	86.4 ± 11.3	68.8–98.1				

ABC Scale, Activities-Specific Balance Confidence scale; Mini-BESTest, Mini Balance Evaluation Systems Tests; 5TSTS, 5 Times Sit-to-Stand Test; 10MWT_Fws, 10-Meter Walk test fast walking speed; 10MWT_Cws, 10-Meter Walk test comfortable walking speed; * statistically significant difference, ^1^ Repeated measures ANOVA/post hoc paired *t*-test, ^2^ Friedmans test/Wilcoxon’s signed rank test.

**Table 3:** Comparisons of limits of stability at 50% and 75% of theoretical limits of stability.

Limits of Stability		Mean ± SD	Time Effect		Mean ± SD	Time Effect	
			F	*p*		F	*p*
Average in four directions ^#^		50% (n = 12)			75% (n = 10)		
RT (s)	Baseline	1.01 ± 0.32			1.02 ± 0.22		
	6 weeks	0.93 ± 0.27	0.455	0.638	0.94 ± 0.13	1.171	0.325
	12 weeks	0.92 ± 0.25			0.93 ± 0.14		
MVL (°/s)	Baseline	3.17 ± 0.75			3.64 ± 0.77		
	6 weeks	3.49 ± 0.91	0.541	0.587	3.79 ± 0.73	0.088	0.916
	12 weeks	3.34 ± 0.50			3.70 ± 0.80		
EPE (%)	Baseline	35.78 ± 8.58			56.85 ± 8.00		
	6 weeks	36.59 ± 10.95	1.227	0.306	57.89 ± 10.87	1.227	0.306
	12 weeks	41.46 ± 8.78			67.87 ± 9.33		
MXE (%)	Baseline	52.27 ± 3.98			74.97 ± 3.46		
	6 weeks	54.09 ± 5.97	0.814	0.452	74.53 ± 2.77	0.105	0.901
	12 weeks	52.00 ± 2.35			75.10 ± 2.48		
DCL (%)	Baseline	33.41 ± 6.74			36.53 ± 9.24		
	6 weeks	33.39 ± 8.28	0.816	0.451	37.83 ± 10.64	0.045	0.956
	12 weeks	36.78 ± 7.34			37.26 ± 9.08		
Towards stroke-affected side		50% (n = 9)			75% (n = 7)		
MVL (%)	Baseline	3.68 ± 0.75			3.81 ± 0.64		
	6 weeks	4.14 ± 1.05	1.198	0.319	3.90 ± 0.86	0.196	0.824
	12 weeks	3.60 ± 0.54			4.06 ± 0.78		
DCL (%)	Baseline	34.16 ± 9.83			36.98 ± 26.43		
	6 weeks	31.58 ± 6.90	0.667	0.523	27.96 ± 11.43	0.398	0.677
	12 weeks	35.96 ± 7.19			32.45 ± 15.59		

DCL, direction control; EPE, endpoint excursion; Four directions, forward, backward, left, right; MVL, movement velocity; MXE, maximal excursion; RT, reaction time; ^#^, forward, backward, left, right.

**Table 4:** Comparison of absolute difference of weight-bearing symmetry and proportion of weight-bearing on the stroke-affected lower limb.

Weight-Bearing Symmetry	Mean ± SD	Time Effect	
		F	*p*
Absolute difference left-right % (n = 12)			
Baseline	6.48 ± 4.62		
6 weeks	6.35 ± 4.36	0.633	0.537
12 weeks	5.03 ± 3.80		
Stroke-affected lower limb % (n = 9)			
Baseline	49.18 ± 4.80		
6 weeks	51.39 ± 4.30	0.646	0.533
12 weeks	50.53 ± 3.22		

## Data Availability

The original contributions presented in this study are included in the article/Supplementary Materials. Further inquiries can be directed to the corresponding author.

## Supplementary Materials

The following supporting information can be downloaded at: https://www.mdpi.com/article/10.3390/healthcare14010129/s1. File S1: Training program S1; File S2: Raw data S2.

## Abbreviations

The following abbreviations are used in this manuscript:

ICF	International Classification of Functioning, Disability and Health
RCT	randomized controlled trial
mini-BESTest	Mini-Balance Evaluation Systems Test
LOS	limits of stability
ABC Scale	Activities-Specific Balance Confidence Scale
10MWT	10-m walk test
5TSTS	5 Times Sit-to-Stand
mPACES	modified Physical Activity Enjoyment Scale
MDC	minimal detectable change
