# Supplementary material for: Group Telerehabilitation to Improve Balance and Mobility in Patients After Stroke Performed at Home: A Feasibility and Pilot Study
Source: Healthcare (Basel). 2026 Jan 4;14(1):129. doi: 10.3390/healthcare14010129 (PMC12785289; doi:10.3390/healthcare14010129)
Supplement: Supplementary file 1 [file healthcare-14-00129-s001.zip › healthcare-4060891-supplementary/RAW DATA_S2_Mocilar et al.pdf]

Supplementary Materials 2:

# Group telerehabilitation to improve balance and mobility in patients after stroke performed at home: A feasibility and pilot study (Močilar et al.)

**RAW DATA**

**Participants' characteristics**

| Participant | Brain stroke hemisphere side | Age (years) | Time post stroke (months) | Weight (kg) | Height (cm) |
|---|---|---|---|---|---|
| 1 | left | 52 | 13 | 84 | 161 |
| 2 | left | 62 | 159 | 81 | 163 |
| 3 | right | 63 | 19 | 90 | 180 |
| 4 | right | 48 | 324 | 85 | 162 |
| 5 | left | 54 | 276 | 61 | 170 |
| 6 | right | 70 | 15 | 67 | 168 |
| 7 | left | 56 | 63 | 83 | 179 |
| 8 | left | 59 | 11 | 61,5 | 163 |
| 9 | left | 48 | 11 | 62 | 162 |
| 10 | right | 58 | 18 | 85 | 168 |
| 11 | right | 54 | 17 | 72 | 173 |
| 12 | right | 61 | 48 | 88 | 172 |
| 13 | left | 43 | 15 | 90 | 182 |

**Adherence**

| Participant | Telerehab. sesions (n) | Video. self-exercise (n) | Video. self-exercise (follow up period) (n) |
|---|---|---|---|
| 1 | 8 | 1 | 7 |
| 2 | 10 | 2 | 5 |
| 3 | 9 | 0 | 10 |
| 4 | 10 | 2 | 0 |
| 5 | 8 | 4 | 10 |
| 6 | 12 | 0 | 10 |
| 7 | 11 | 0 | 0 |
| 8 | 10 | 2 | 0 |
| 9 | 11 | 0 | 0 |
| 10 | 12 | 0 | 12 |
| 11 | 9 | 0 | 0 |
| 12 | 12 | 0 | 0 |
| 13 | 11 | 1 | 12 |

**mPACES**

| Participant | I enjoy - do not enjoy it (points 1-7) | I dislike - like it (points 1-7) | It is no fun - is fun (points 1-7) | My body feels good - does not feel good (points 1-7) | It frustrates me - does not frustrate me (points 1-7) |
|---|---|---|---|---|---|
| 1 | 1 | 7 | 7 | 1 | 7 |
| 2 | 1 | 7 | 6 | 2 | 7 |
| 3 | 1 | 7 | 6 | 1 | 7 |
| 4 | 1 | 7 | 7 | 1 | 7 |
| 5 | 1 | 7 | 6 | 1 | 6 |
| 6 | 1 | 7 | 7 | 1 | 2 |
| 7 | 3 | 7 | 5 | 4 | 6 |
| 8 | 1 | 7 | 7 | 1 | 7 |
| 9 | / | / | / | / | / |
| 10 | 1 | 7 | 7 | 1 | 1 |
| 11 | 2 | 7 | 7 | 1 | 6 |
| 12 | 3 | 5 | 4 | 2 | 7 |
| 13 | 2 | 6 | 5 | 1 | 7 |

PRIMARY OUTCOME MEASURES

**miniBESTest**

| Participant | Baseline (points) | After training (points) | Six weeks after training (points) |
|---|---|---|---|
| 1 | 22 | 22 | 23 |
| 2 | 20 | 20 | 21 |
| 3 | 14 | 15 | 18 |
| 4 | 14 | 15 | 16 |
| 5 | 15 | 21 | 22 |
| 6 | 14 | 17 | 15 |
| 7 | 16 | 21 | 20 |
| 8 | 17 | 22 | 20 |
| 9 | 21 | 23 | / |
| 10 | 14 | 15 | 18 |
| 11 | 22 | 27 | 27 |
| 12 | 20 | 23 | 24 |
| 13 | 21 | 26 | 26 |

**5TSTS**

| Participant | Baseline (s) | After training (s) | Six weeks after training (s) |
|---|---|---|---|
| 1 | 14,6 | 15,2 | 15,79 |
| 2 | 13,6 | 8,8 | 10,42 |
| 3 | 15,2 | 12,6 | 8,43 |
| 4 | 17,5 | 12,2 | 13,89 |
| 5 | 11 | 9,7 | 9,78 |
| 6 | 19,2 | 13,1 | 13,83 |
| 7 | 14,1 | 14 | 13,83 |
| 8 | 38,4 | 16,6 | 15,96 |
| 9 | 13,9 | 12,5 | / |
| 10 | 20 | 13,1 | 17,01 |
| 11 | 10,4 | 9,1 | 8,64 |
| 12 | 12,2 | 12,7 | 12,3 |
| 13 | 18,2 | 14,2 | 12,8 |

**10MWT_Comfortable walking speed**

| Participant | Baseline (m/s) | After training (m/s) | Six weeks after training (m/s) |
|---|---|---|---|
| 1 | 0,85 | 0,88 | 0,96 |
| 2 | 1,05 | 1,2 | 1,24 |
| 3 | 0,83 | 0,92 | 0,97 |
| 4 | 1 | 0,91 | 0,99 |
| 5 | 0,99 | 1,07 | 1,16 |
| 6 | 1,03 | 1,03 | 1,07 |
| 7 | 1,38 | 1,14 | 1,53 |
| 8 | 1,18 | 1,22 | 1,29 |
| 9 | 1,2 | 1,29 | / |
| 10 | 0,94 | 1,02 | 0,92 |
| 11 | 1,35 | 1,27 | 1,26 |
| 12 | 1,11 | 1,08 | 0,96 |
| 13 | 1 | 1,18 | 1,26 |

**10MWT_Fast walking speed**

| Participant | Baseline (m/s) | After training (m/s) | Six weeks after training (m/s) |
|---|---|---|---|
| 1 | 1,22 | 1,37 | 1,62 |
| 2 | 1,52 | 1,59 | 1,56 |
| 3 | 1,2 | 1,29 | 1,5 |
| 4 | 1,28 | 1,2 | 1,25 |
| 5 | 1,47 | 1,69 | 1,67 |
| 6 | 1,26 | 1,39 | 1,26 |
| 7 | 2,08 | 2 | 2,02 |
| 8 | 1,23 | 1,5 | 1,61 |
| 9 | 1,58 | 1,88 | / |
| 10 | 1,16 | 1,14 | 1,3 |
| 11 | 1,9 | 2,07 | 2,11 |
| 12 | 1,72 | 1,7 | 1,69 |
| 13 | 1,33 | 1,55 | 1,55 |

SECONDARY OUTCOME MEASURE

**LOS 50 %**

| Participant | Baseline | | | | | After training | | | | | Six weeks after training | | | | |
|---|---|---|---|---|---|---|---|---|---|---|---|---|---|---|---|
| | RT (s) | MVL (°/s) | EPE (%) | MXE (%) | DCL (%) | RT (s) | MVL (°/s) | EPE (%) | MXE (%) | DCL (%) | RT (s) | MVL (°/s) | EPE (%) | MXE (%) | DCL (%) |
| 1 | 1,65 | 2,63 | 28,72 | 51,66 | 38,27 | 0,76 | 2,70 | 48,70 | 57,07 | 30,14 | 1,10 | 2,71 | 48,50 | 51,12 | 39,61 |
| 2 | 0,74 | 3,78 | 38,82 | 51,55 | 34,13 | 0,60 | 4,71 | 42,62 | 59,55 | 31,49 | 0,80 | 3,31 | 32,02 | 48,70 | 44,21 |
| 3 | 1,26 | 2,95 | 29,51 | 52,66 | 41,53 | 0,92 | 3,41 | 31,24 | 49,65 | 32,90 | 0,83 | 3,27 | 37,07 | 51,69 | 34,04 |
| 4 | 1,10 | 3,04 | 35,90 | 51,82 | 28,85 | 0,72 | 3,44 | 29,70 | 53,21 | 42,98 | 0,78 | 3,48 | 45,93 | 52,36 | 28,43 |
| 5 | 0,72 | 2,78 | 32,76 | 51,23 | 35,77 | 0,98 | 3,16 | 33,62 | 51,56 | 40,14 | 0,65 | 3,39 | 50,37 | 52,28 | 40,57 |
| 6 | 1,40 | 3,98 | 47,77 | 57,40 | 26,93 | 0,47 | 5,02 | 18,89 | 51,14 | 28,14 | 1,00 | 3,87 | 34,29 | 52,27 | 27,53 |
| 7 | 0,53 | 2,79 | 34,16 | 53,94 | 36,50 | 1,17 | 2,46 | 25,56 | 46,26 | 48,92 | 1,07 | 3,02 | 36,22 | 50,73 | 48,83 |
| 8 | 1,03 | 2,43 | 30,70 | 44,19 | 25,78 | 1,36 | 2,40 | 35,56 | 45,80 | 34,11 | 1,53 | 2,70 | 45,59 | 51,01 | 33,47 |
| 9 | 0,79 | 4,60 | 44,99 | 58,80 | 27,23 | 0,65 | 4,96 | 45,43 | 55,65 | 31,03 | / | / | / | / | / |
| 10 | 0,84 | 2,55 | 32,42 | 50,81 | 43,91 | 1,25 | 4,13 | 30,16 | 61,76 | 20,93 | 0,91 | 3,61 | 55,05 | 57,91 | 29,82 |
| 11 | 0,72 | 3,73 | 24,99 | 50,76 | 29,45 | 0,78 | 2,95 | 37,00 | 56,59 | 28,18 | 0,63 | 3,48 | 50,85 | 54,65 | 35,99 |
| 12 | 1,15 | 4,88 | 55,74 | 60,66 | 22,03 | 1,01 | 4,60 | 57,09 | 64,91 | 22,95 | 1,04 | 4,45 | 36,57 | 51,21 | 31,28 |
| 13 | 1,06 | 2,55 | 37,82 | 50,57 | 37,73 | 1,09 | 2,88 | 48,98 | 51,61 | 39,83 | 0,67 | 2,83 | 25,05 | 50,10 | 47,57 |

**LOS 75 %**

| Participant | Baseline | | | | | After training | | | | | Six weeks after training | | | | |
|---|---|---|---|---|---|---|---|---|---|---|---|---|---|---|---|
| | RT (s) | MVL (°/s) | EPE (%) | MXE (%) | DCL (%) | RT (s) | MVL (°/s) | EPE (%) | MXE (%) | DCL (%) | RT (s) | MVL (°/s) | EPE (%) | MXE (%) | DCL (%) |
| 1 | 1,32 | 2,86 | 62,08 | 72,84 | 50,95 | 1,03 | 3,26 | 66,26 | 71,73 | 47,83 | 1,03 | 3,26 | 66,26 | 71,73 | 47,83 |
| 2 | 0,84 | 3,58 | 62,63 | 75,99 | 31,53 | 0,84 | 4,01 | 68,09 | 75,23 | 39,15 | 0,84 | 4,01 | 68,09 | 75,23 | 39,15 |
| 3 | / | / | / | / | / | / | / | / | / | / | / | / | / | / | / |
| 4 | 1,14 | 3,94 | 57,13 | 71,99 | 35,89 | 0,97 | 3,31 | 44,43 | 68,73 | 26,25 | 0,97 | 3,31 | 44,43 | 68,73 | 26,25 |
| 5 | 1,09 | 3,11 | 67,88 | 74,38 | 32,25 | 1,09 | 3,34 | 71,18 | 75,38 | 36,56 | 1,09 | 3,34 | 71,18 | 75,38 | 36,56 |
| 6 | 0,93 | 4,58 | 51,34 | 79,53 | 38,28 | 0,79 | 5,12 | 46,15 | 76,48 | 19,94 | 0,79 | 5,12 | 46,15 | 76,48 | 19,94 |
| 7 | 1,28 | 3,35 | 56,27 | 72,41 | 36,55 | 1,11 | 3,22 | 67,84 | 73,71 | 49,17 | 1,11 | 3,22 | 67,84 | 73,71 | 49,17 |
| 8 | / | / | / | / | / | / | / | / | / | / | / | / | / | / | / |
| 9 | / | / | / | / | / | / | / | / | / | / | / | / | / | / | / |
| 10 | 0,91 | 3,10 | 63,69 | 77,15 | 43,16 | 1,02 | 3,79 | 42,31 | 76,89 | 45,48 | 1,02 | 3,79 | 42,31 | 76,89 | 45,48 |
| 11 | 0,68 | 4,02 | 39,71 | 71,75 | 27,33 | 0,96 | 3,42 | 59,59 | 75,60 | 48,50 | 0,96 | 3,42 | 59,59 | 75,60 | 48,50 |
| 12 | 0,77 | 5,13 | 52,41 | 81,49 | 20,92 | 0,71 | 5,04 | 51,75 | 78,22 | 25,37 | 0,71 | 5,04 | 51,75 | 78,22 | 25,37 |
| 13 | 1,21 | 2,77 | 55,41 | 72,17 | 48,46 | 0,85 | 3,35 | 61,34 | 73,33 | 40,04 | 0,85 | 3,35 | 61,34 | 73,33 | 40,04 |

**Weight-bearing symmetry**

| Participant | Baseline | | After training | | Six weeks after training | |
|---|---|---|---|---|---|---|
| | Left (%) | Right (%) | Left (%) | Right (%) | Left (%) | Right (%) |
| 1 | 52,02 | 47,98 | 52,88 | 47,12 | 52,28 | 47,7 |
| 2 | 51,11 | 48,89 | 51,46 | 48,54 | 51,71 | 48,3 |
| 3 | 45 | 55 | 41,39 | 58,61 | 48,61 | 51,4 |
| 4 | 59,11 | 40,89 | 47,49 | 52,51 | 45,43 | 54,6 |
| 5 | 45,49 | 54,51 | 44,78 | 55,22 | 43,6 | 56,4 |
| 6 | 53,59 | 46,41 | 53,89 | 46,11 | 52,01 | 48 |
| 7 | 51,48 | 48,52 | 53,61 | 46,39 | 49,17 | 50,8 |
| 8 | 47,02 | 52,98 | 49,33 | 50,67 | 49,91 | 50,1 |
| 9 | 48,64 | 51,36 | 49,54 | 50,46 | / | / |
| 10 | 48,56 | 51,44 | 47,93 | 52,07 | 49,75 | 50,3 |
| 11 | 50,8 | 49,2 | 46,85 | 53,15 | 47,74 | 52,3 |
| 12 | 43,96 | 56,04 | 44,52 | 55,48 | 46,88 | 53,1 |
| 13 | 52,66 | 47,34 | 51,27 | 48,73 | 55,27 | 44,7 |

**ABC Scale**

| Participant | Baseline (%) | After training (%) | Six weeks after training (%) |
|---|---|---|---|
| 1 | 77,81 | 75,63 | 82,19 |
| 2 | 91,25 | 92,50 | 91,25 |
| 3 | 92,50 | 93,75 | 89,69 |
| 4 | 71,75 | 61,56 | 70,63 |
| 5 | 93,44 | 95,63 | 95,94 |
| 6 | 71,56 | 85,63 | 90,63 |
| 7 | 78,75 | 72,19 | 70,63 |
| 8 | 56,56 | 83,13 | 83,13 |
| 9 | 98,75 | 100,00 | / |
| 10 | 46,88 | 51,25 | 68,75 |
| 11 | 96,56 | 97,50 | 98,50 |
| 12 | 89,38 | 95,94 | 97,81 |
| 13 | 79,38 | 94,38 | 98,13 |

**Legend**

mPACES - modified Physical Activity Enjoyment Scale

MiniBESTtest - Mini-Balance Evaluation Systems Test

5TSTS - 5 times sit to stand

10mWT - 10 meter walk test

/ - missing data

LOS 50 % - Limits of stability at 50 % theoretical limit of stability

LOS 75 % - Limits of stability at 75 % theoretical limit of stability

RT - reaction time

MVL - movement velocity

EPE - endpoint excursion

MXE - maximum excursion

DCL - direction control

ABC Scale - Activities-Specific Balance Confidence Scale
