# Supplementary material for: Group Telerehabilitation to Improve Balance and Mobility in Patients After Stroke Performed at Home: A Feasibility and Pilot Study
Source: Healthcare (Basel). 2026 Jan 4;14(1):129. doi: 10.3390/healthcare14010129 (PMC12785289; doi:10.3390/healthcare14010129)

Supplementary Materials 1:

Group telerehabilitation to improve balance and mobility in patients after stroke performed at home: A feasibility and pilot study (Močilar et al.)

Training program

WARM-UP (4.5 minutes)

1. Seated marching (1.5 minutes)

Instructions	Sit upright on the chair and lift your feet alternately and symmetrically off the floor as you march.
Progression	2. higher knee lifts<br>3. higher knee lifts and shoulder circles forwards (45 seconds) and backwards (45 seconds)<br>4. higher knee lifts and shoulder circles in the opposite direction

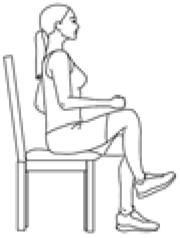

2. Half squats (1.5 minutes)

Instructions	Stance position. Support yourself with both hands on the back of the chair and do squats. Make sure your back is straight and that your knees are moving towards your feet.
Progression	2. support with one hand on the chair<br>3. without support<br>4. changing speed (15 seconds slow, 15 seconds fast etc.)

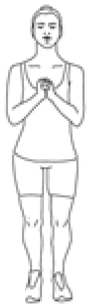

3. Marching in place (1.5 minutes)

Instructions	Stand with one hand supported on the chair and march on the spot, distributing your weight evenly on both legs and lifting your knees symmetrically.
Progression	2. without support<br>3. without support, and alternating lifts of the upper limbs towards the ceiling (5 times each side) and to the side (5 times each side)<br>4. without support, and alternating lifts of the upper limb towards the ceiling and to the side with a water bottle in each hand

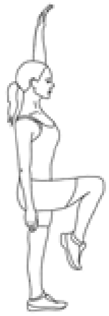

MAIN SESSION (40–50 minutes)

1. Forward reach (5 times) and diagonal reach (5 times)

The goals	Weight-bearing, improve weight shifting, improve sitting, strengtl core muscles, improve dynamic balance.
Instructions	Sit with the upper limbs extended forward and fingers interlocked, lean forward with a straight back and then diagonally to feel the weight shift to the feet.
Progression	2. arms parallel without fingers interlocked<br>3. with a half-liter water bottle, holding with both hands<br>4. with two half-liter water bottles, one in each hand

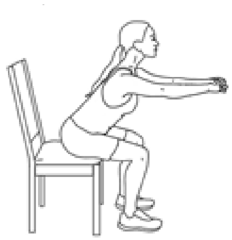

2. Walks with the buttocks

The goals	Preparation for standing up, strengthening the core muscles
Instructions	Place the unaffected hand on your chest and try not to move it to the left and right. Start sitting at the end of the chair and shorten one side of your torso by alternately lifting your hips and moving your buttocks backwards. When you have reached the back of the chair, repeat the movement forwards to the edge of the seat. Repeat this 5 times.
Progression	2. two sets of 5 repetitions<br>3. three sets of 5 repetitions<br>4. three sets of 8 repetitions

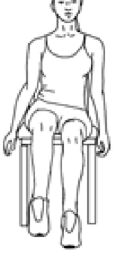

**3. Simultaneous elbow flexion standing (three sets, 10 repetitions)**	
The goals	Strengthening the elbow flexor muscles, improve static balance in stance position.
Instructions	Stand and simultaneously bend your upper limbs and touch your shoulders with palms of your hands.
Progression	2. one half-liter water bottle in both hands<br>3. two half-liter water bottles in one hand each, from a position with flexed elbows into extension with a flexion of 90°in the shoulders<br>4. alternate punching with two half-liter water bottles

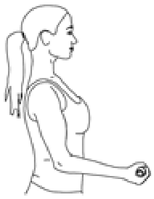

**4. Stretching the elbow, wrist and finger flexors (30 seconds per side)**	
The goals	Stretching and normalizing muscle tone of the elbow, wrist, and finger flexors to prevent muscle contractures.
Instructions	Place your palm and fingers on a flat surface (e.g. table, drawer), extend your elbow and lean gently on the hand.
Progression	External rotation of the shoulder joint and greater dorsiflexion of the wrist.

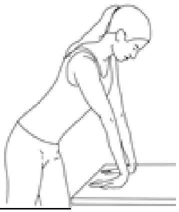

**5. Standing up and sitting down on a chair (three sets, five repetitions)**	
The goals	Standing up and sitting down on a chair safely, independently, and as symmetrically as possible, strengthening the lower limb muscles, improving dynamic balance, increasing speed of standing up and sitting down
Instructions	First sit on the chair with your hips, knees, and feet in the same plane. The feet are aligned. Stand up with your weight evenly distributed on both legs and straighten your legs.
Progression	2. Three sets of 10 repetitions with upper limbs crossed on the chest<br>3. Three sets of 12 repetitions with upper limbs crossed on the chest and alternating tempo (3 fast, 3 slow, 3 fast, 3 slow)<br>4. Three sets of 10 repetitions with asymmetrical foot position (the affected lower limb is placed behind the healthy lower limb)

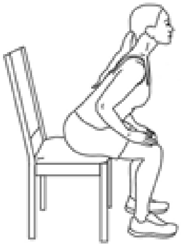

**6. Weight shift while standing**	
The goals	Improvement of dynamic and proactive balance.
Instructions	Hold on to the chair in front of you with the unaffected hand and shift the weight in splits to the left foot and then to the right foot (5 times on each side), then place the feet hip-width apart and shift the weight forward and then backward (5 times each). Move only in the ankles without breaking the body in the hips.
Progression	2. without support, shift the weight faster, and hold the end position (3 seconds)<br>3. without support, sideways/forwards/backwards in the direction of movement with the upper limb<br>4. without support, with half-liter water bottles in the hands and reaching in the direction of movement

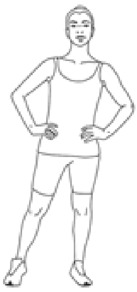

**7. Standing with a reduced base of support (30 seconds)**	
The goal	Improving static balance.
Instructions	Step with the affected leg in front of the unaffected leg so that the toes of the unaffected foot touch the heel of the affected foot. Shift your weight evenly onto both feet and hold the position for 30 seconds (if necessary, hold on to the armrest of the chair). Then change foot position and hold for a further 30 seconds.
Progression	2. stand on one leg, other leg in the air (30 seconds per position)<br>3. stand on one leg and move the other leg forwards and backwards (30 seconds per position)<br>4. stand on one leg and move the other leg medially (in front of the standing leg) and laterally

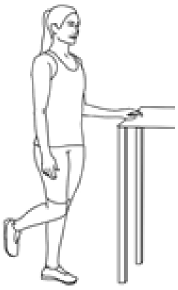

**8. Toe raises (three sets, 10 repetitions)**	
The goals	Improve the ability to safely lift the toes without support, improve the endurance and strength of the plantar flexor muscles and improve dynamic balance.
Instructions	Hold on to the armrest of the chair with one hand, stand on your toes on both sides with equal weight on your feet and slowly lower yourself onto your heels. Repeat 10 times.
Progression	2. toe raises on both sides with alternating speed (3 slow raises, 3 fast raises, etc.)<br>3. bilateral fast toe raises (3 sets, 8 repetitions per leg)<br>4. unilateral toe raises (3 sets, 8 repetitions)

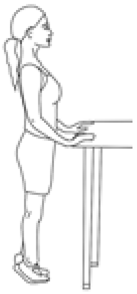

**9. Standing position; upper limb elevation through anteflexion (3 sets, 12 repetitions)**	
The goals	Strengthening the shoulder and shoulder girdle muscles and improving balance while standing.
Instructions	Stand with your feet hip-width apart and interlace the fingers of your hands. Slowly raise your arms upwards without arching your back and then lower your arms to the starting position.
Progression	2. with a half-liter water bottle in your hands<br>3. with a half-liter water bottle in your hands and alternating speed (3 slow, 3 fast), the head follows the bottle (flexion and extension)<br>4. with two half-liter water bottles and feet together

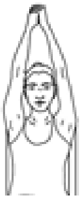

**10. Foot movements in different patterns (5 repetitions per leg)**	
The goals	Improve dynamic balance and coordination as well as weight shift on the affected lower limb.
Instructions	Stand on the unaffected leg and take a step forward with the affected leg, then a lateral step, then a lateral step and a step backwards, then a lateral step and a cross-steps. If necessary, hold on to the armrest of the chair. Now stand on the affected leg.
Progression	2. weight shift with the stepping leg<br>3. weight shift with the stepping leg and lunge with the other lower limb<br>4. cha cha cha (two steps on the spot and two steps to the side)

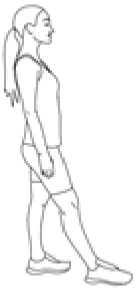

**11. Squats (three sets, 12 repetitions)**	
The goals	Strengthening the lower limb muscles, improve weight shift on the affected lower limb, improve dynamic balance and changing position.
Instructions	Stand with your feet hip-width apart, hold on to the armrest of the chair, and squat down as if you were sitting on the chair. Make sure that the weight is evenly distributed on both legs and pay attention to the movement of the knees, which must go in the direction of the toes.
Progression	2. without support<br>3. without support and with alternating speed (3 slow, hold the final position for 2 seconds, 3 fasts, etc)<br>4. alternate between squatting, sitting, and standing on the chair (6 repetitions)

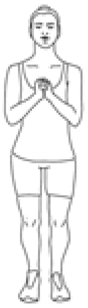

COOL DOWN (5 minutes)

**1. Seated (2 minuti)**	
Instructions	Sit upright on the chair and lift your feet alternately and symmetrically off the floor.
Progression	2. higher knee lifts<br>3. higher knee lifts and shoulder circles forwards (1 minute) and backwards (1 minute)

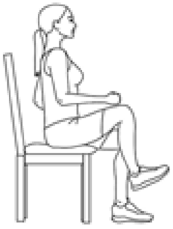

2. Knee flexor muscles stretch (30 seconds each leg)

Instructions	Sit on the edge of the chair, stretch out one leg and bend your foot towards you. Lean forward with your back straight, looking at your foot, until you feel a stretch in your thigh muscles and hold the position.

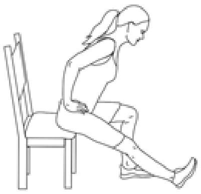

3. External hip rotator muscles stretch (30 seconds each leg)

Instructions	Sit down, lift the ankle of one leg and place it on the knee of the other leg. Grasp the ankle with your hands and lean forward, pushing the leg outwards with your elbow on the side of the raised leg. Hold the position when you feel a stretch in the buttocks of the raised leg.

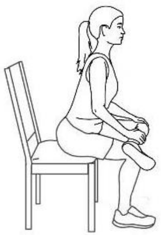

4. Calf muscles stretch (30 seconds each leg)

Instructions	Stand up, step one leg back and straighten the knee. The heel of the back leg should remain on the floor while you move the pelvis over the front leg and shift the weight. Hold the position when you feel a stretch in the calves.

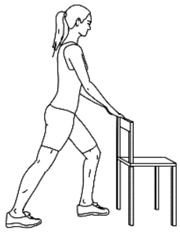

Supplement: Supplementary file 1 [file healthcare-14-00129-s001.zip › healthcare-4060891-supplementary/Training program_S1_Mocilar et al.pdf]
